# Learning My Way: A Pilot Study of Navigation Skills in Cerebral Palsy in Immersive Virtual Reality

**DOI:** 10.3389/fpsyg.2020.591296

**Published:** 2020-11-30

**Authors:** Emilia Biffi, Chiara Gagliardi, Cristina Maghini, Chiara Genova, Daniele Panzeri, Davide Felice Redaelli, Anna Carla Turconi

**Affiliations:** Scientific Institute, IRCCS Eugenio Medea, Bosisio Parini, Italy

**Keywords:** cerebral palsy, visual-spatial, immersive virtual reality, navigation skills, children

## Abstract

**Purpose:** Human navigation skills are essential for everyday life and rely on several cognitive abilities, among which visual-spatial competences that are impaired in subjects with cerebral palsy (CP). In this work, we proposed navigation tasks in immersive virtual reality (IVR) to 15 children with CP and 13 typically developing (TD) peers in order to assess the individual navigation strategies and their modifiability in a situation resembling real life.

**Methods:** We developed and adapted to IVR an application based on a 5-way maze in a playground that was to be navigated to find a reward. The learning process, navigation strategies, and adaptation to changes were compared between participants with CP and their TD peers and correlated with visual-spatial abilities and cognitive competences.

**Results:** Most participants with CP needed more attempts than TD participants to become proficient in navigation. Furthermore, the learning phase was correlated to visual-spatial memory but not with cognitive competences. Interestingly, navigation skills were comparable between groups after stabilization. While TD participants mainly relied on allocentric strategies based on environmental cues, egocentric (self-centered) strategies based on body motion prevailed in participants with CP. Furthermore, participants with CP had more difficulties in modifying their navigation strategies, caused by difficulties in executive processes beyond the visual-perceptual impairment, with an inefficient shift between implicit and explicit competences.

**Conclusions:** The navigation abilities in participants with CP seem to be different from their TD peers in terms of learning and adaptation to new conditions; this could deeply affect their everyday life and ultimately participation and inclusion. A regular assessing and focused rehabilitative plans could help to better navigate the environment and affect self-perception.

## Introduction

Cerebral palsy (CP) is one of the leading causes of disabilities in children in western countries, affecting 1–2.5 per 1,000 live births (Reid et al., 2011). The term CP includes a heterogeneous spectrum of nonprogressive brain disorders manifesting with motor, sensory, and cognitive deficits (Rosenbaum et al., 2007). Even if motor impairment often represents the most remarkable manifestation of the disorder, disturbances of sensation, perception, cognition, communication, and behavior commonly affect the patients’ quality of life.

Impairments in visual-spatial competences and spatial organization are common in CP (Pirila et al., 2004; Stiers and Vandenbussche, 2004; Gagliardi et al., 2013), and they are assessed mainly by well-known standardized tests such as the Corsi Block Test (Orsini et al., 1987). Nevertheless, few studies focused on navigation abilities and their relation with visual-spatial abilities in subjects with CP (Pavlova et al., 2006; Belmonti et al., 2015b; Berthoz and Zaoui, 2015).

Spatial navigation refers to the ability to maintain a sense of direction/location while moving around in the environment in order to find one’s way. It includes abilities such as orienting in complex environments, perceiving distance and planning routes to distant locations as well as mentally representing the reciprocal relations of landmarks in space (Lawton, 2010; Wolbers and Hegarty, 2010; van der Ham et al., 2020). Several cognitive abilities underpin navigation skills, including long-term memory (Spiers and Maguire, 2008), executive functions (Lithfous et al., 2013; Ruggiero et al., 2016), precision in encoding multisensorial (visual, vestibular, and proprioceptive) experiences, as well as the ability to form mental representations used to guide behavior (Bianchini et al., 2014).

Alternative navigation strategies may be used to travel the space. On the one hand, self-based or egocentric strategy (hereafter ES) relies mainly on gradually learned and egocentric representations (based on body parts) and tends to be based mainly on (implicit) procedural memory. On the other hand, allocentric strategy (AS), i.e., the world-based navigation, is grounded mainly on flexible, allocentric representations, relative to external objects or environmental features and depends more on (explicit) declarative memory (Tolman, 1948; Berthoz, 1997; Gillner and Mallot, 1998; Lind et al., 2013; Belmonti et al., 2015a; Negen et al., 2018).

Human navigation abilities largely vary across subjects, change with age, and develop gradually in time (Siegel and White, 1975; Lehnung et al., 2003; van der Ham et al., 2020), from ES to AS during childhood, that goes along with the ability to combine perceptual experience into a unitary representation (Siegel and White, 1975; Acredolo, 1978). The cognitive mapping knowledge develops gradually and is not fully functioning until the age of 10 years (Lehnung et al., 1998). As described by Ruggiero et al. (2016), AS and ES have different developmental trends. With respect to AS, processing times improve significantly along with age (and decline in elders), while accuracy is less affected by age. ES shows an inverse pattern, where accuracy improves and processing times are less affected by age. Overall, when directly performing navigation tasks, there is a change in performance around 7 years old, with an increased use of AS (Bullens et al., 2010). Efficient adult navigation would require allocentric as well as egocentric encoding with a need of flexibility and ability to switch between different referent frames, thus underlining the crucial role of executive functions as determinant of navigation skills along development (Purser et al., 2012).

Navigation skills rely on the integrity of the spatial neural network, which includes occipital, parietal, frontal, and temporal lobes – hippocampus and parahippocampal cortex, retrosplenial and postero-cingulate cortex [see (Boccia et al., 2014) for a meta-review for fMRI studies]. As described by Galati et al. (2010), and Belmonti et al. (2015a), ES encoding is limited to the dorsal visual stream, while AS involves also the ventral visual stream.

Among the few studies on navigation ability in CP (Pavlova et al., 2006; Belmonti et al., 2015b; Berthoz and Zaoui, 2015), Pavlova et al. (2006) described an impairment in visual navigation in adolescents with periventricular leukomalacia (PVL) in a cognitive task, which did not require body navigation. The authors related the performance at a paper-and-pencil task derived from the Labyrinth of the WISC-III (Wechsler Intelligence Scale for Children, third edition) to the locomotor performance and to the neuroimaging data, thus showing the involvement of frontal lesions in the navigation impairment and of the parietal periventricular white matter in visual processing in children with CP.

To assess navigation abilities in developmental age, Belmonti et al. (2015b) developed the Magic Carpet, a task derived from the Corsi Block Test to study navigation in body space thanks to an electronic device. They confirmed the impairment in visual spatial memory (as assessed by the Corsi Block Test) in participants with CP with respect to typically developing (TD) controls, while they did not find such differences in their navigation task.

New paradigms for studying visual-spatial function and spatial memory strategies in children with CP were described by Berthoz and Zaoui (2015). They also proposed the use of non-immersive Virtual Reality (VR) to study spatial navigation with paradigms combining visuo-manual and locomotor tasks.

Indeed, non-immersive VR systems have been increasingly used in children and adults to assess navigation skills (Iglói et al., 2009; Bullens et al., 2010; Cogne et al., 2017, 2018; Coutrot et al., 2018; Leon et al., 2018; Negen et al., 2018; van der Ham et al., 2020). In particular, Iglói et al. (2009) developed a pc-based adaptation of the StarMaze to examine the learning strategies in a dynamic situation. Their pc-based pentagonal maze with five radiating arms was to be navigated through in first-person perspective and using a joystick, in order to achieve a specific goal (find a treasure). They studied spontaneously adopted navigation strategies in adults as well as their ability to shift from one to another strategy when needed. By proposing the same task, Bullens et al. (2010) delineated the developmental time course of the sequential egocentric and allocentric representations in children, advancing the paradigm in which the spontaneous or imposed use of ES/AS could be assessed.

To offer a more ecological and fully controlled setting, thanks to the replication of a “real” environment, where the user tends to use all of the assumptions about how things work in the real world (Bowman et al., 2002), immersive VR (IVR) systems can be adopted. These platforms are equipped with large projection screens and motion tracking systems (e.g., cave), or head mounted displays (HMD) or flat screens with 3D glasses, and are a technological revolution for the understanding of mental processes (Freeman et al., 2017). IVR is an ideal tool to study human spatial navigation: participants can perceive and act upon a very life-like interactive environment (van Veen et al., 1998). However, only few studies have used IVR to assess spatial navigation, and they were mainly focused on aging and dementia (Zakzanis et al., 2009; Freeman et al., 2017; Leon et al., 2018).

The aim of the present work is to explore the navigation and learning strategies adopted by children and preadolescents with CP and to compare them with those adopted by their TD peers in an ecological situation in IVR, where the participants had to navigate the environment by moving their body in the space.

**Figure 1:**
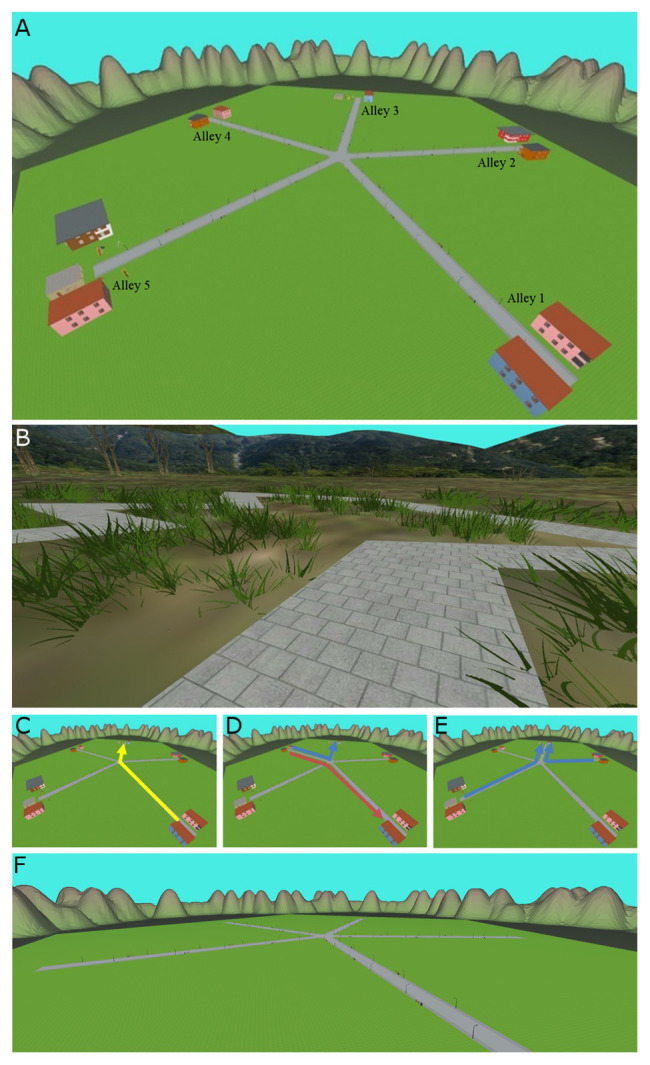
Immersive Virtual Reality (IVR) scenarios and tasks. **(A)** The Star maze scene developed for the GRAIL system. **(B)** The “Walk along the pathway” environment used in the training phase. **(C)** Path to be followed during the training trials in Task 1 (yellow path). **(D)** Allocentric (blue path) and egocentric (red path) navigation during test trials in Task1. **(E)** Two paths imposed during compelled allocentric trials (blue paths). **(F)** The bare maze used in the Task 2‐ compelled ES.

We developed and adapted to IVR, the maze task as previously proposed for computer by Iglói et al. (2009). Our task was proposed in a playground IVR environment (Figure 1A) to participants with CP and their TD peers; the participant had to navigate the maze in order to find a “treasure” at the end of an alley. The reward was visible only when found without any explicit communication about the conditions of the task given to the participant. A “free strategy” task let the participant free to adopt ES or AS and to switch between them during learning and testing trials. The flexibility of the navigation skills was explored with two additional sets of trials, where the reward could be located in one position reachable only by ES or only by AS, thus imposing to the participant to modify his/her preferred strategy. Participants explored the maze standing on a dynamic platform based on IVR (Motek GRAIL system – see below in section “Materials and Methods” for details). A semi-dark external environment led to a more realistic situation, driving the participants to focus their attention on the virtual environment projected on the surrounding 180-degree screen, while avoiding interfering stimuli coming from elsewhere.

Given the well-known impairment in visual-spatial skills, we expected the participants with CP to perform worse than their TD peers at visual-spatial pen-and-paper task and to navigate the IVR maze differently. More specifically, we hypothesized that participants with CP would be less efficient in learning to navigate and would have more difficulties in integrating external landmarks and therefore in navigating by using AS, as assessed by the test in IVR.

## Materials and Methods

### Participants

Patients were recruited in the Neuro-rehabilitation Unit of the Scientific Institute IRCCS E. Medea in Italy. The inclusion criteria were diagnosis of bilateral CP; age between 6 and 14 years old; severity of motor impairment classified in level I, II, and III, according to Gross Motor Function Classification System (GMFCS; Palisano et al., 1997) and Manual Ability Classification System (MACS; Eliasson et al., 2006); and ability to follow the instructions. The exclusion criteria were as follows: severe muscle spasticity and/or contracture, a diagnosis of severe learning disability, behavioral problems, and visual difficulties that would affect the proposed activity and participation (i.e., Snellen Visual Acuity <3/10”).

According to these criteria, 15 participants suffering from bilateral CP were recruited (11 males, four females, mean age 10.3 ± 2.2 years old), classified as to GMFCS I/II/III: 11/0/4 and as to MACS I/II/III: 10/4/1 (Table 1).

**Table 1:** Demographic features of participants.

	CP	TD	*p*
Gender M/F	11/4	5/8	0.063^&^
Age (years)^$^	10.4 ± 2.2	10.4 ± 2.3	0.936^&&^
GMFCS I/II/III	10/0/4	-	
MACS I/II/III	10/4/1	-	
RAVEN Z-score^£^	0 (2.5)	2.5 (2)	**0.008** ^&&&^

M, males and F, females. Significant values of *p* are in bold.

$ Mean and standard deviation.

£ Median and interquartile range.

& value of *p* of the chi-square test for uniformity.

&& value of *p* of the unpaired *t*-test.

&&& value of *p* of the Mann-Whitney U test.

A control group of TD peers was included to have a reference in the navigation task. Thirteen TD participants were recruited (five males, eight females, mean age 10.4 ± 2.3 years; Table 1). All TD participants were healthy, with no history of psychiatric or neurological illness, learning disabilities, and hearing or visual loss; they showed average school performances in language, arts, and reading.

Raven’s Colored Progressive Matrices (Lezak et al., 2004) were administered to all participants after the recruitment to assess general cognitive competences. No particular adaptation of standard test conditions was necessary, apart from postural adaptations.

The Ethics Committee of the Scientific Institute approved the study protocol. Written informed consent to participate in this study was provided by each participants’ legal guardian/next of kin in accordance with the Declaration of Helsinki. The study has been registered as a clinical trial on ClinicalTrials.gov (NCT04270305).

### Spatial Abilities

Spatial abilities were assessed by two classical paper-and-pencil tests (the Corsi Block Test and the Labyrinth) proposed before the navigation task in IVR.

The Corsi Block Test (Orsini et al., 1987) assesses visuospatial memory and consists of a set of identical wooden blocks arranged are not aligned on a desktop. The tester taps on a number of blocks in sequence, and the participant is asked to reproduce the tapped sequence, which is of variable length.

The Labyrinth subtest of the WISC-III (Wechsler Intelligence Scale for Children, third) consists of 10 paper-and-pencil tasks with increasing complexity (Weschler, 1991). The participant has to find the way out from the center of a two-dimensional maze; this test measures planning ability, perceptual organization, visual-motor coordination, and self-control.

### Virtual Environment

#### The GRAIL System

The GRAIL (Gait Real-time Analysis Interactive Lab system, by Motek NL) is a system, which integrates IVR environments projected on a 180° cylindrical screen, a Vicon motion-capture system (Oxford Metrics, Oxford, United Kingdom) and a two-degree of freedom platform. The patient is thus immersed in virtual environments and can naturally walk (see for instance (Gagliardi et al., 2018) for a thorough description of the system). The whole system is controlled by the D-flow software (Motek NL), which regulates the relationships between the participant, the scenario, and interactive feedbacks (Geijtenbeek et al., 2011). The D-flow software was used to develop an application that traces the positions of two reflective markers placed on the posterior superior iliac spines of the subject, by means of the motion-capture system, and lets the participant navigate within the virtual environment accordingly. Furthermore, the D-flow was used to implement object collisions and specific events. Specifically, when the subject moves forward or backward, his/her movement within the IVR environment accelerates or decelerates, respectively; when he/she shifts the pelvis right or left, and he/she turns right or left within the IVR scene. Furthermore, the application activates specific events when the participant reaches specific positions within the IVR environment.

#### The Maze

We developed an interactive star maze task in IVR adapted from a five-arm maze used in other studies (Iglói et al., 2009; Bullens et al., 2010). The interactive maze consisted of a playground with environmental cues (e.g., swings, slides, houses, and mountains) placed between the ends of adjacent alleys. Five alleys radiated from the angles of a regular pentagon in the center of the maze (Figure 1A). For the creation of the GRAIL scene, objects (i.e., environmental cues) and scenario were individually modeled by means of Google SketchUp. Then, files were exported in the COLLADA format and imported into Autodesk 3ds Max software, to be assembled within the scenery. The final scene, containing the environment and the individual objects, was exported in Ogre format and finally uploaded within the D-flow software. At the end of each alley of the maze, different landmarks were designed (e.g., houses with different shapes and colors and the presence/absence of a swing).

### Navigation Experimental Procedure in IVR

Immediately prior to testing, the participant underwent a “Walk along the pathway” training phase to accustom participants to the GRAIL system and to the movements necessary to control the application. He/she had to walk along a zigzag pathway, to rotate and come back, paying attention not to go off the road. This training lasted up to 10 min until the participant was able to follow the way (Figure 1B). If the participant was able to complete the training, he/she went on to the navigation test, otherwise they were not included in the test.

The experimental procedure was made of two tasks (Task 1 and Task 2 described below), with a duration of 45 min for each participant. The IVR tasks were proposed in the maze described in section “The Maze” and participants were asked to navigate the maze to find a “treasure” at the end of an alley, invisible until reached. Each navigation lasted up to 2 min, with a stable verbal instruction “look for the treasure” despite the variations of conditions that were never explicitly disclosed to the participant.

#### Task 1: Free Navigation

Each participant carried out 21 explorations of the maze, with 16 attempts to freely explore the environment (training sessions) plus five interposed trials used to assess the preferred strategy (Table 2). The same starting point (alley 1) and location of the reward (end of alley 3) were maintained in the 16 training sessions (Figure 1C). During the five “assessing” trials, the exploration started from a different point (alley 4), and the reward was placed both in alley 3 and in alley 1. This meant that the participant could find the treasure either in alley 3 with AS (using the environmental cues previously learnt) or in alley 1 with ES (using the previously learnt body motions), as shown in Figure 1D.

**Table 2:** Details of the trials in Task1 (“Free strategy”).

Trial number	Starting point	Treasure location	Trial type	Training trial number	Test trial number
1	Alley 1	Alley 3	Training	1	
2	Alley 1	Alley 3	Training	2	
3	Alley 1	Alley 3	Training	3	
4	Alley 1	Alley 3	Training	4	
5	Alley 1	Alley 3	Training	5	
6	Alley 4	Alley 1, Alley 3	Test		1
7	Alley 1	Alley 3	Training	6	
8	Alley 1	Alley 3	Training	7	
9	Alley 1	Alley 3	Training	8	
10	Alley 4	Alley 1, Alley 3	Test		2
11	Alley 1	Alley 3	Training	9	
12	Alley 1	Alley 3	Training	10	
13	Alley 1	Alley 3	Training	11	
14	Alley 4	Alley 1, Alley 3	Test		3
15	Alley 1	Alley 3	Training	12	
16	Alley 1	Alley 3	Training	13	
17	Alley 4	Alley 1, Alley 3	Test		4
18	Alley 1	Alley 3	Training	14	
19	Alley 1	Alley 3	Training	15	
20	Alley 1	Alley 3	Training	16	
21	Alley 4	Alley 1, Alley 3	Test		5

**Figure 2:**
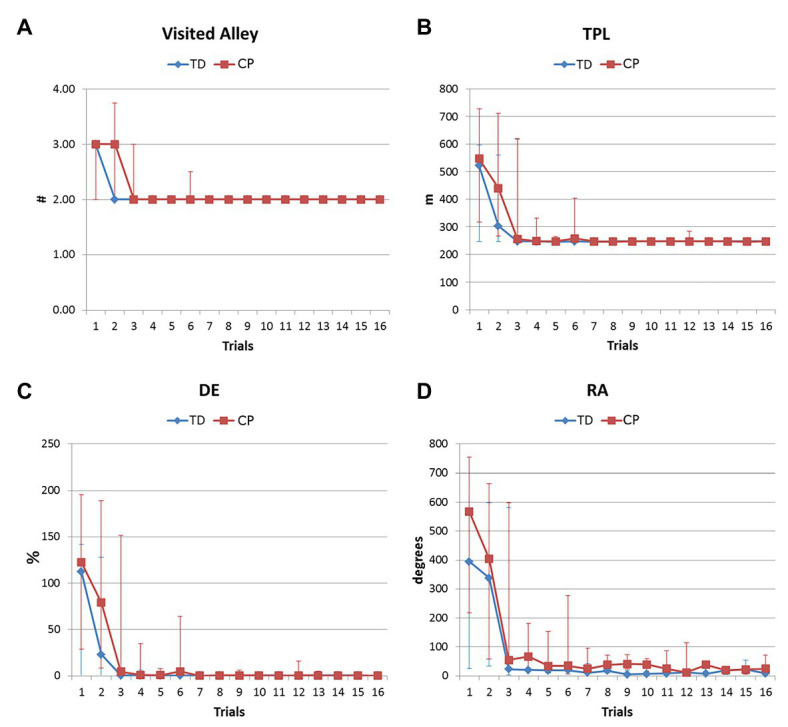
Navigation skills and modifications during the training in the free strategy task. Learning curves of TD subjects (blue) and participants with CP (red) were obtained during 16 training sessions. **(A)** Number of visited alleys (the correct number is 2); **(B)** total path length (the minimum is 246 m); **(C)** distance error %, and **(D)** rotation angle. Median values and interquartile ranges are shown.

*Trial number* refers to the trial order, independently from the trial type (training or test). *Training trial number* enumerates only training trials, as used in Figures 2, 4. *Test trial number* counts only test trials.

#### Task 2: Compelled Strategies

In these sessions, each participant was forced to navigate the maze using alternatively AS or ES to locate the reward. There were four “compelled AS” trials and three “compelled ES” trials, as done in (Iglói et al., 2009). During the “compelled AS” trials, the navigation started from a new alley (2 and 5, twice each) and the reward was placed in alley 3; participants were not aware of the different starting point. The treasure could thus be reached only using AS: the body movements used to find the treasure in Task 1 (Figures 1C,D) indeed differed from those needed to find the treasure in compelled AS (Figure 1E). Therefore, participants had to use environmental cues. Conversely, in the three “compelled ES” trials, the participant navigated from alley 1 and the reward was located in alley 3, as in Task 1, but the maze was bare (Figure 1F). Only the five alleys, the grass, and the surrounding mountains were visible, and the participants were forced to reproduce previously performed sequence of body turns. The sequence of the items (compelled AS and compelled ES) was counterbalanced.

### Data Analysis and Statistics

#### Demographic Data and Spatial Abilities

Z scores were computed for Raven’s Colored Progressive Matrices (Raven Z score hereinafter), Corsi Block Test (Corsi Z score hereinafter), and Labyrinth Test from WISC-III (Labyrinth Z score hereinafter).

Demographic data were compared between CP and TD groups with unpaired tests. Specifically, the number of males and females was compared by means of the chi-square test for uniformity, the age of participants (normally distributed) was compared with the unpaired t-test, while the Mann-Whitney U test was used to evaluate differences in the Raven Z scores (since the distribution was not normal, as verified by a Lilliefors test). Similarly, data describing spatial abilities and sequence learning competences were compared by means of the Mann-Whitney U test. Normally distributed data are reported in the results section as mean (standard deviation-SD), while not normally distributed data are shown as median (interquartile range-IQR; data shown in Tables 1 and 3).

**Table 3:** Visual spatial competences in CP and TD participants.

	CP	TD	*p*^&^
Corsi block test Z-score	-0.4 (1.2)	0.7 (1.8)	**0.021**
Labyrinth subtest Z-score	-0.7 (2.3)	1.3 (1)	**0.003**

Median values and interquartile ranges are shown. Significant values of *p* are in bold.

& values of *p* of the Mann–Whitney U test.

### IVR Navigation Task

During the navigation, the participant’s position in the virtual environment as well as his/her movements were recorded by the GRAIL application within a Cartesian coordinate system. Success/no success in reaching the reward in the given time (2 min) was computed for each attempt.

For each navigation trial, we computed the following parameters by a custom-made software developed in Matlab (Mathworks):

- success/no success in finding the reward (yes/no),
- number of visited alleys to get the treasure (#),
- total traveled road (Total Path Length, TPL ‐ m),
- time needed for each navigation (s), and
- mean speed (m/s).

As formerly done in Bullens et al. (2010), we computed two parameters to qualify the efficiency of the navigation skills. The higher the value, the worse the performance in both parameters.

- Distance Error (DE, Equation 1), which evaluates the total distance traveled by the subject to find the treasure with respect to the minimum distance needed following an ideal pathway.

(1) $$\mathrm{DE}\;\left(\%\right)=100*\left(\begin{array}{l} \mathrm{total distance}\\ \mathrm{traveled}-\mathrm{ideal}\\ \mathrm{distance traveled} \end{array}\right)/\mathrm{ideal distance traveled}$$

- Rotation Angle (RA, Equation 2) that assesses the number of rotations (i.e., the lateral movements used to look around when you are in the center of the maze) of the subject in relation to minimum number of rotations necessary to follow an ideal pathway. The greater the rotation value, the more participants had “looked around” in the environment by making turns with their body.

(2) $$\mathrm{RA}\;\left(\mathrm{degrees}\right)=\mathrm{Participant's}\;\mathrm{rotations}-\mathrm{minimum rotations}$$

#### Task 1: Free Navigation

During free navigation, the number of trials required to succeed in learning the way through the virtual maze within the 16 training trials (parameter success/no success) was evaluated for each subject. Specifically, we counted the number of participants that

- were able to get the reward from the first trial and never failed subsequently,
- found the treasure after the first and before the third trial,
- succeeded in finding the way before the seventh trial (but after the third excluded), and
- never obtained a stable success.

The time course of the number of visited alleys, the TPL, the DE, and the RA within the 16 learning navigations allowed following the learning process in the navigation of IVR maze for each participant. In order to identify the attempt from which the performance became stable for participants with CP and their TD peers, we looked for the “stabilization trial,” that is the indicator that the participants found his/her way to navigate the maze. To do that, navigation performance (i.e., median values within each group, in every training trial, of visited alleys, TPL, DE, and RA) in the 16 trials was plotted. All the four curves featured a “knee” (see section “Results”) that is the point, where the curve visibly bends, specifically from high slope to low slope (flat or close to flat). To verify the accuracy of the value of this “knee” (that was identified as “stabilization trial”), two tests were performed on the four datasets: a paired non-parametric repeated measure test (Friedman test) among all the data after the “knee” (that should be comparable, if the data are stable after the knee) and a paired non-parametric Wilcoxon test between the “knee” and the preceding value (that should be different). Then, navigation performance at the “stabilization trial” (i.e., the knee) were compared between TDs and participants with CP by means of a Mann-Whitney U test, and they were correlated with Raven Z score by means of a non-parametric correlation analysis.

To evaluate if visual-spatial abilities and cognitive competences influence the learning when navigating in a new environment, a correlation analysis was performed. Specifically, the median values of number of visited alleys, TPL, DE, and RA over the trials before the stabilization were computed and a non-parametric correlation analysis between these data and Corsi Z score, Labyrinth Z score, and Raven Z score was run.

Moreover, the strategy freely adopted by each participant in each of the five “assessing” trial was determined considering, which alley they reached. The strategy was defined as follows:

- AS when the participant reached the end of Alley 3, thus using the environmental cues previously learnt (and by entering only two alleys the starting alley and the final one), and
- ES, when the participant found the treasure at the end of Alley 1, thus using the previously learnt body-based strategies and entering only two alleys (the starting alley and the final one),
- No efficient strategy otherwise (e.g., no success in finding the treasure or more than 2 alleys visited).

Afterward the five assessing trials were evaluated as a whole for each participant, who was classified as follows:

- Allocentric, if he/she used an AS in more than three consecutive assessing trials (and the others were no efficient trials),
- Egocentric, if he/she used ES in more than three consecutive assessing trials (and the others were no efficient trials),
- Shifter, if he/she used both ES and AS along test trials, and
- Lacking of efficient strategy, when more than three trials were classified as no efficient.

Afterward learning curves were evaluated again taking into account the strategy used by each participant.

#### Task 2: Compelled Strategy

We considered successful only trials with a direct route from the starting point to the alley with the reward, without wandering in other alleys (only two visited alleys). For each participant, the percentage of success in reaching the reward was calculated separately for the “forced” allocentric and egocentric navigations.

Afterward median values (and IQR ranges) for the whole group and for the ES and AS subgroups (as defined in Task 1) were computed for the “forced” AS and ES.

All the statistical analyses were carried out with IBM SPSS Statistics v21 and the significance level was set at 5%. To evaluate the power of the analyses, it was estimated how likely it was to observe a significant effect in the computed parameters, given the sample size of 13 TD participants vs. 15 participants with CP, an expected large effect size (Cohen’s *d* = 1) and the α level set at 0.05. The software G Power 3 was used for this estimation.

## Results

Demographic details of participants are shown in Table 1. The CP and TD groups were comparable except for a statistically significant difference in terms of Raven’s Colored Progressive Matrices, with participants with CP performing worse (Z-score CP 0 (2.5), TD 2.5 (2), *p* = 0.008), though in normal range. Similarly, TD participants performed better than participants with CP in the spatial tasks [Corsi Z score: TD 0.7 (1.8), CP −0.4(1.2), *p* = 0.021; and Labyrinth Z score: TD 1.3 (1), CP −0.7(2.3), *p* = 0.003], as shown in Table 3.

All the participants were able to complete the “Walk along the pathway” training and be included in the navigation test. Considering Task 1 and the parameter success/no success, all 13 TD participants succeeded in learning the way through the virtual star maze from the first trial and never failed subsequently. On the other hand, only seven participants with CP (less than 50%) succeeded similarly to TDs, while four participants with CPs succeeded within the third trial and maintained the success in the following trials, three participants succeeded stably from the eighth trial; and one participant never succeeded stably.

Beyond success, the navigation performance in the virtual maze improved along with trials in both groups. Figure 2 shows learning curves for participants with CP (red line) and TD (blue line), considering number of visited alley, total path length, distance error, and rotation angle. The knee of the curve, after which the characteristics of the performance became stable, was identified in the fifth trial for the TD group (minimum *p*-value for the Friedman test =0.513 and maximum value of *p* for the Wilcoxon test =0.033) and in the seventh trial for participants with CP (minimum value of *p* for the Friedman test =0.143; maximum value of *p* for the Wilcoxon test =0.036) for all the considered parameters. Even if participants with CP stabilized later than TDs, the performance in the two groups did not differ significantly when stable (fifth trials for TD, seventh for CP: minimum value of *p* for the Mann Whitney U test =0.201), as shown in Table 4. Furthermore, the performance at the “stabilization trial” (i.e., the knee) was not correlated to the Raven Z-score (all *p* > 0.311, all Spearman’s rho<0.198).

**Table 4:** Data at the stable trial for CP (seventh trial) and TD (fifth trial) participants.

	CP	TD	Th value	*p*^&^
Visited alley (#)	2 (0)	2 (0)	2	0.964
TPL (m)	246.9 (2.7)	246.6 (0.79)	246	0.363
DE (%)	0.37 (1.12)	0.26 (0.33)	0	0.339
RA (°)	25.2 (78.9)	18.9 (28.3)	0	0.201
Mean speed(ms^−1^)	10.5 (5.2)	9.4 (5)	-	0.618
Time(s)	30 (25.7)	30.5 (14.7)	-	0.821

The fourth column (*Th value*) reports the theoretical best value for each parameter. TPL, total path length; DE, distance error; and RA, rotation angle. Median (interquartile) values are reported.

& *p*-value of the Mann-Whitney U test.

Navigation skills along the learning process before the “knee” (i.e., considering four trials in TD participants and six trials in participants with CP) during the Free Strategy Task (Task 1) correlated with Corsi Z score [specifically with TPL −0.419(0.026), DE −0.419(0.026) and RA −0.473(0.011), expressed as Spearman’s rho (*p* value), but not with Raven Z score (all *p* >0.514) as shown in Table 5.

**Table 5:** Results of the correlation analysis between visual spatial competences and cognitive abilities with characteristics of the performance during learning phase (i.e., four trials for TD participants, six trials for participants with CP).

	Visited alleys	TPL	DE	RA
Corsi block test Z-score	−0.280 (0.149)	−0.419 (**0.026**)	−0.419 (**0.026**)	−0.473 (**0.011**)
Labyrinth subtest Z-score	−0.119 (0.546)	−0.217 (0.267)	−0.217 (0.267)	−0.270 (0.165)
RAVEN Z-score	−0.121 (0.541)	−0.076 (0.701)	−0.076 (0.701)	−0.129 (0.514)

Data are reported as Spearman’s rho (*p*-value). Significant values of *p* are in bold.

**Figure 3:**
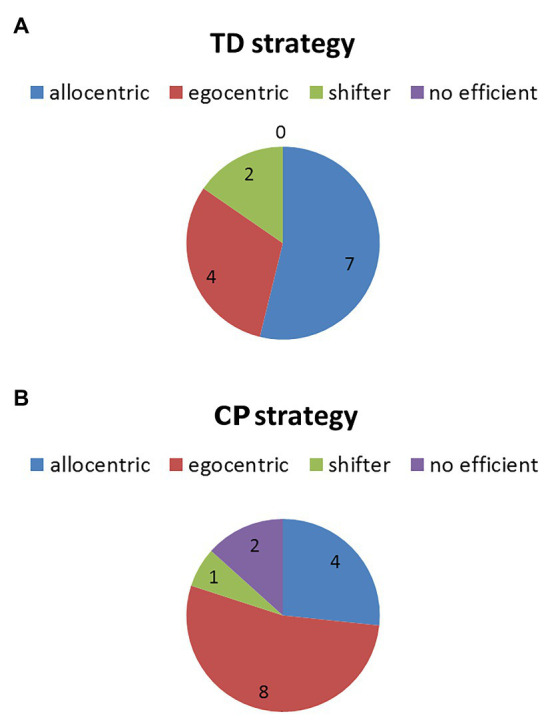
Pie chart representing the distribution of the strategies freely adopted by **(A)** TD subjects and **(B)** participants with CP.

Results about the strategy freely adopted by each participant during the five test trials are presented in Figure 3. The AS strategy was adopted by 54% of TD and 27% of participants with CP; the ES strategy was chosen by 31% of TDs and 53% of CPs; and 15% of TDs and 7% of CPs were shifter while 13% of CPs had a no efficient strategy.

**Figure 4:**
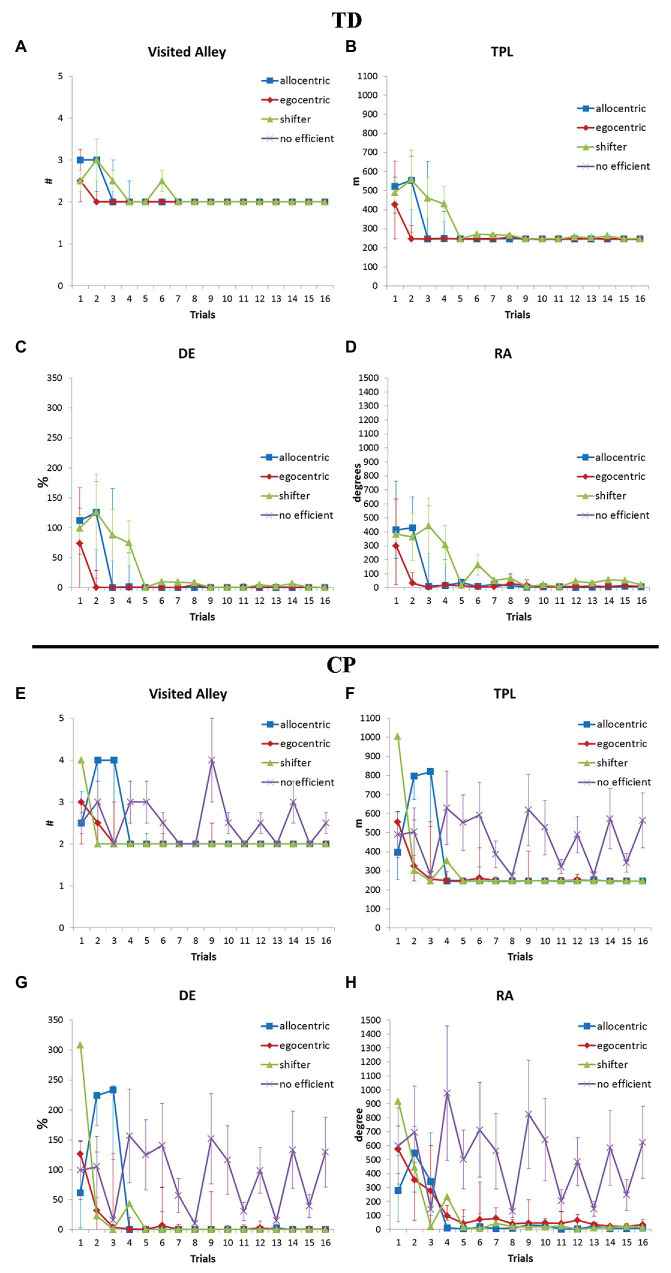
Differences in navigation skills (number of visited alleys; TPL, total path length; DE, distance error %; and RA, rotation angle) and modifications during the training according to the freely adopted strategy in TD **(A–D)** and participants with CP **(E–H)**. Error bars represent interquartile ranges.

Figure 4 shows learning curves of TD and CP participants, grouped by the strategy used in the test trials. In both groups, the faster learning was reached with ES; in TD group, AS seemed to be related to a faster learning than the shifter one but this was reversed in the CP group; the absence of a strategy led to an inefficient performance, characterized by instability of all parameters.

**Figure 5:**
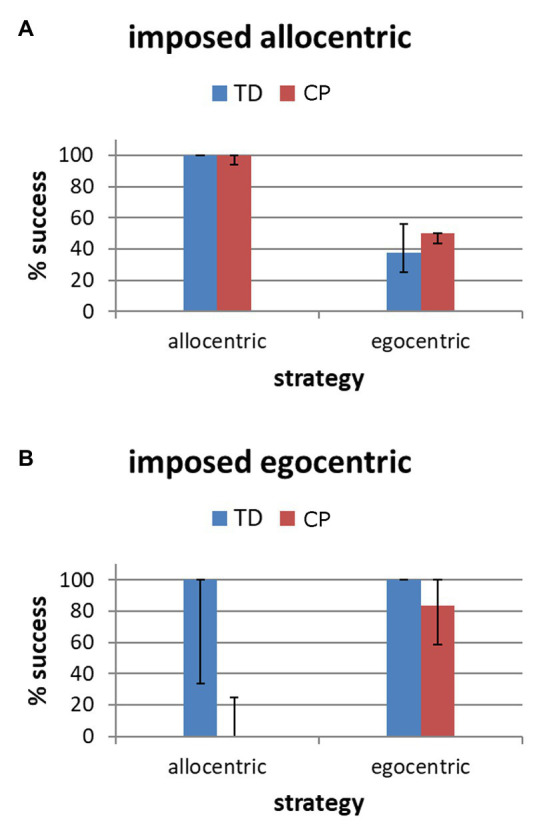
Success (express as percentage) in the trials with imposed allocentric **(A)** and egocentric **(B)** strategies in CP and TD participants, considering AS and ES subgroups (as defined by freely adopted strategies).

Finally, when participants were compelled to modify strategies (Task 2), TD participants showed a tendency to perform better than participants with CP in the imposed allocentric trials [TD 100(25), CP 50(25), *p* = 0.081], while the performance was similar in the egocentric trials [TD 100(33.3), CP 66.7(83.3), *p* = 0.189], though with high variability mainly in the CP group. Considering subgroups, participants of both groups with AS performed well in the allocentric compelled trials (Figure 5A, left columns); similarly, participants with ES of both groups had good performance in the egocentric compelled trials (Figure 5B, right columns). TD participants with AS succeeded in the shift to ES, while AS participants with CP failed (Figure 5B, left columns). On the other hand, participants with ES of both groups were similarly impaired in the shifting to AS (Figure 5A, right columns).

Considering the sample size of 13 TD participants vs. 15 participants with CP, the *α* level set at 0.05 and an expected large effect size (Cohen’s *d* = 1), and the probability to observe a significant effect, i.e., the estimated power, resulted equal to 0.82, that is usually considered a quite satisfactory value.

## Discussion

In this paper, we focused on studying navigation behavior of participants with CP and their TD peers in conditions similar to “real life” thanks to a maze task to be navigated in IVR. The navigation in IVR involves the movement of the whole body in space and is subtended by processes, where memory (implicit and explicit), representation, and ongoing modifications at multiple levels (perceptual, sensorial, and motorial) determine the performance similarly to real life. In the immersive virtual environment, the participant has to encode interrelationships among environmental landmarks, movements, and the location of the goal while moving the whole body, as in real life. As far as we know, no study on this topic was performed in an IVR setting.

By this pilot study, we meant to highlight similarities and differences between CP young participants and their TD peers in terms of navigation skills in IVR and their relation with visual-spatial skills as assessed by standardized pen-and-paper tests.

TD participants succeeded immediately in finding their way in the maze independently from the strategy they preferred, reached in few attempts their most efficient navigation performance and consolidated their learning. AS was preferred by half TDs and ES by one third of them, with the remaining TDs being shifters. When compelled to modify their strategy, those TD participants who had been spontaneously using AS were able to efficiently navigate also with ES, thus demonstrating to be able to shift to sequential sensory/motorial integration and efficiently perform also without landmarks. The shift from the spontaneous ES to the compelled AS was challenging for half of ES TDs, who failed to reach the target, evidencing a difficulty to rapidly account for landmarks and spatial relations. AS is an highly-demanding online integration of the characteristics of the surround, and the process – especially as to extrapersonal space – is age sensitive (Ruggiero et al., 2016). Though our TD group is too small to describe a reliable developmental trajectory, our data could be compatible with the spontaneous use of AS emerging along school years up to adulthood with ES persisting, as presented by (Laurance et al., 2003; Iglói et al., 2009), and with 10-year-old children performing similar to adults according to Bullens et al. (2010). Our TD participants who were capable to navigate with AS could also navigate with ES if needed; half of those who relied mainly on ES were not able to resort to AS or, at least, to efficiently inhibit the implicitly learnt strategy.

In the CP group, the performance was somehow different and less homogeneous. Half of participants with CP succeeded immediately in identifying the way similarly to their TD peers, though they needed more attempts to navigate efficiently (seven attempts instead of five as in TD participants). Most of other participants with CP were slower both in identifying and in reaching their best navigation performance, except for one who failed to learn stably his/her way. Interestingly, navigation skills along the learning process correlated with visual-spatial abilities but not with cognitive competences. Nevertheless, participants with CP navigated similarly to their TD peers when the performance was consolidated, without significant impact of the motor impairment. Differently from TDs, most participants with CP adopted ES to navigate but showed similar difficulties to shift to AS if compelled. Moreover, participants with CP had more difficulties in shifting also from AS to ES when compelled.

Even if we must carefully consider that our groups were small, different hypothesis could be put forward to explain these preliminary findings. AS asks for efficient online visual-spatial integration, beyond memory skills and representation. A dorsal visual stream vulnerability has been described in bilateral CP as in other neurodevelopmental disorders affecting visual-spatial-motor integration (Atkinson and Braddick, 2011). The connectivity among those systems is crucial, with potential increasing difficulties in participants with CP to resort to information encoded in both visual streams (dorsal and ventral) as in allocentric navigation. In our study, most TDs but few participants with CP (about 25%) used allocentric navigation. The difficulty to adopt AS – both spontaneously and if compelled – could therefore share some pathways with the visual-spatial impairment well-described in CP (Pirila et al., 2004; Pagliano et al., 2007), and thus be linked to the brain organization in CP. A confirmation to this hypothesis could come from the correlation we found between the performance at a visual-spatial task (as Corsi Block Test) and the learning phase of navigation.

On the other hand, though the two groups were similar with respect to their average age, most participants with CP could have adopted ES as if they were younger, with a sort of developmental delay with respect to their TD counterparts (even if we did not find relation of navigation skills with cognitive competences in either group). Though a developmental delay could not be completely excluded, an atypical and not a simply delayed performance could probably be more compatible with the presence of the brain injury in CP. As stated by (D’Souza and Karmiloff-Smith, 2017), the initial impairment could lead to “*cascading effects*” on other systems, which could develop atypically under different constraints.

The possibility to modify the strategy when needed offers further topics in this direction. Modifying the strategy and resorting to AS was difficult also for TD participants adopting ES, while the reverse was easier. Moreover, participants with CP adopting AS were totally unable to resort to ES when needed, differently from TDs. Therefore, navigation strategies in CP, although sufficiently efficient when trained, were rigidly fixed to the way (pathway) they were learnt along. If it were just a delay issue in a similar developmental trend, the shift from AS to ES should have been possible in participants with CP just as in their TD peers. This was not the case, corroborating the “atypical” hypothesis (D’Souza and Karmiloff-Smith, 2017) for most participants with CP.

Navigation skills and visual-spatial abilities are interrelated but do not coincide, because several cognitive abilities are required during navigation. In order to move around to get a target, visual information must be online integrated with sensory/motor complex perception; additionally, a proper planning of the route in a complex spatial framework and an ongoing verification must take place concurrently. Moreover, memory is deeply involved during navigation tasks, both at explicit and implicit level. In our task, the presence of interposed test trials along the task interfered with the learning process and asked for the adaptation of the strategy to the modified conditions.

The key difference between participants with CP and their TD peers could therefore lie in the efficiency of the **dynamic** process of learning and adapting to modifications, much more than in the “**effectiveness**” of the performance once stabilized. Were it true, any time something new has to be learnt, the process could be more problematic in participants with CP than in their TD peers. Indeed, participants with CP were not able to modify their strategy in a few attempts and persisted along the previously learnt route. The difficulty did not seem to pertain only to the need of processing and integrating more complex visual-spatial data, but also to shift between implicit and explicit modalities, to inhibit or modify behavior, together with a “problem solving” modified situation: in brief, it seemed to imply also an impairment in executive functions, previously described in subjects with CP (Sakash et al., 2018). Interestingly, when the learning was attained, the performance of both groups was not related to the traditional spatial tasks, similarly to a previous study with a different task (Belmonti et al., 2015b).

The depicted pattern of subjects with CP could deeply affect learning, everyday life, and ultimately participation and inclusion. In real life situation, when a modification in the environment takes place or more data have to be integrated or the consolidated strategy fails, most individuals with CP could not cope with modifications and they would continue navigating with greater difficulty than their TD peers. Moreover, as a part of participants with CP failed to learn to successfully navigate, subjects with CP probably need to follow a different way to learn to navigate their space, where explicit instructions and step-by-step strategies could be putatively more effective than implicit learning. This should be considered in order to identify possible targets for rehabilitation of subjects with CP.

Though limited by the small number of participants, our pilot study offers a contribution not only to improve the knowledge of spatial competences but also to individuate potential treatments and rehabilitation intervention for individuals with CP. Among the multiple targets of intervention proposed in this condition, navigation skills are seldom considered even if crucial in everyday life. Our preliminary data suggest to take into account the ability to navigate especially in dynamic and new situations, when flexibility and simultaneous online processing are required. This could be done from childhood and may help to develop autonomy to move around efficiently in the surrounding environment. But not only moving around is implied. Spatial and social behavior are intertwined, as stated in a recent paper “Who we are is where we are” (Proulx et al., 2016): our minds map our bodies and the world around us. ES and AS in navigation as well as individual differences in cognitive and sensory abilities could impact on how we map the environment and ourselves in the environment. Thus, being able to efficiently navigate could go beyond movement and become strictly linked to social skills and role (Pasqualotto and Proulx, 2012, 2013). At the same time, properties of the social and physical environment can reciprocally impact on the individual, inviting different strategies in navigation, and eventually result in different perspectives of the self in relation to the environment. An effort to help children and preadolescents with CP to better navigate could therefore ultimately impact on their self-perception and ability to interact with their physic and social environment as they grow up.

IVR could play a role, helping to create controlled environments, where different variables can be manipulated and real life-like conditions can be offered. Two possible pathways for rehabilitation could derive from our data. Firstly, a targeted training could help in sustaining adaptability in individuals with CP, in order to cope with the environment modifications in everyday life when moving. Secondly, a targeted intervention could be planned for those subjects with CP who have more difficulties to find and learn their way, supposedly relying more on explicit strategies.

The present work has some limitations. First, the relatively small number of recruited patients asks for caution in generalizing our results, which need to be confirmed in larger samples. However, the results highlighted the feasibility and validity of the approach and detected different navigation strategies in the recruited sample of participants with CP. A possible consequence of a small sample size is a low power of data analysis. In this pilot study, we did not conduct an *a priori* sample size definition; in contrast we computed the probability to observe a significant effect that means we estimated a *post hoc* power, considering to observe a large effect in our data; this estimated power was acceptable (>0.8). Nevertheless, statistically significant differences between participants with CP and their TD peers were assessed in the dataset.

Another limitation is that the two groups were unbalanced in terms of gender, with more males in the CP group, even if the excess was not statistically significant. Sex differences are well-documented in spatial abilities, in which men on average outperform women (Linn and Petersen, 1985) with and interaction between gender and sex hormones (Pletzer et al., 2019). However, a recent paper from van der Ham et al. (2020) investigating navigation abilities in 11,000 of healthy subjects has demonstrated that navigation skills have no gender effect in children (aged 8–17), while a male advantage is present for the adult population.

In conclusion, our navigation tasks in IVR was suitable to study the learning phase of navigation skills, to verify the efficacy of navigation after learning and to evaluate the possibility to modify the navigation strategies when needed, in participants with CP and in their TD peers.

Future works will explore navigation skills in larger samples and will be balanced in terms of gender. Moreover, they will focus on the rehabilitation of these abilities in clinical practice. This ground competence indeed affects mobility and is involved in motor planning as well as in cognitive and social skills. A better understanding could significantly improve the clinical outcome of patients with CP.

## Data Availability Statement

The datasets presented in this study can be found in online repositories. The names of the repository/repositories and accession number(s) can be found at:

http://doi.org/10.5281/zenodo.4110824.

## Ethics Statement

The studies involving human participants were reviewed and approved by Ethic committee of Scientific Institute, IRCCS Eugenio Medea. Written informed consent to participate in this study was provided by the participants’ legal guardian/next of kin.

## Author Contributions

CGa and AT designed the research project. EB, CGe, DR, CM, and DP developed the scenario, the acquisition of data, and recruited the participants. EB analyzed the data. CGa, AT, and EB interpreted the data and prepared the paper. All authors contributed to the article and approved the submitted version.

### Conflict of Interest

The authors declare that the research was conducted in the absence of any commercial or financial relationships that could be construed as a potential conflict of interest.
